# Valvular Cardiomyopathy: The Value of Cardiovascular Magnetic Resonance Imaging

**DOI:** 10.1155/2022/3144386

**Published:** 2022-02-22

**Authors:** Vasiliki Tsampasian, Sandeep S. Hothi, Thuwarahan Ravindrarajah, Andrew J. Swift, Pankaj Garg, Vassilios S. Vassiliou

**Affiliations:** ^1^Norwich Medical School, University of East Anglia, Norwich Research Park, Norwich, UK; ^2^Norfolk and Norwich University Hospital, Norwich, UK; ^3^The Institute of Cardiovascular Sciences, College of Medical and Dental Sciences, University of Birmingham, Birmingham, UK; ^4^Department of Infection, Immunity and Cardiovascular Disease, University of Sheffield, Sheffield, UK

## Abstract

Cardiovascular magnetic resonance (CMR) imaging has had a vast impact on the understanding of a wide range of disease processes and pathophysiological mechanisms. More recently, it has contributed significantly to the diagnosis and risk stratification of patients with valvular heart disease. With its increasing use, CMR allows for a detailed, reproducible, qualitative, and quantitative evaluation of left ventricular volumes and mass, thereby enabling assessment of the haemodynamic impact of a valvular lesion upon the myocardium. Postprocessing of the routinely acquired images with feature tracking CMR methodology can give invaluable information about myocardial deformation and strain parameters that suggest subclinical ventricular impairment that remains undetected by conventional measures such as the ejection fraction (EF). T1 mapping and late gadolinium enhancement (LGE) imaging provide deep myocardial tissue characterisation that is changing the approach towards risk stratification of patients as an increasing body of evidence suggests that the presence of fibrosis is related to adverse events and prognosis. This review summarises the current evidence regarding the utility of CMR in the left ventricular assessment of patients with aortic stenosis or mitral regurgitation and its value in diagnosis, risk stratification, and management.

## 1. Introduction

Valvular heart disease (VHD) has a high prevalence worldwide with mild disease affecting up to one in two people over the age of 65 [1, 2]. A substantial number of individuals in the primary care setting with symptoms of heart failure suffer from clinically significant VHD, most commonly aortic stenosis and mitral regurgitation [3]. Undoubtedly, prompt diagnosis and appropriate management are vital in positively influencing the prognosis and future course of the disease.

Traditionally, and still dominating much of cardiovascular medicine now, valvular heart disease assessment, and myocardial function has relied heavily upon echocardiographic data. Over recent years, as our knowledge and understanding of the pathophysiological mechanisms underlying VHD expands, it has become apparent that perhaps assessing the severity of valvular disease alone without the impact this has on the myocardium may be insufficient to guide prognostication and explain consequent VHD-related cardiomyopathy, morbidity, and mortality. This is evident in patients with similar degrees of valvular stenosis, but differences in clinical presentation and outcomes that relate to myocardial dysfunction. In this respect, cardiovascular magnetic resonance (CMR) has an invaluable role as, with its high spatial resolution, it allows accurate qualitative and quantitative assessment of left ventricular wall thickness, mass, volumes, and ejection fraction, which are of high prognostic value and thus allows accurate assessment, diagnosis, and risk stratification.

CMR has proved to successfully fill in gaps and answer both scientific and clinical questions, not only because it can provide a detailed evaluation of the valve function and anatomy but also because it can assess the haemodynamic consequences of the valvular lesions on the myocardium that is directly associated with the valve. T1 mapping and late gadolinium enhancement (LGE) allow direct assessment of myocardial fibrosis and changes in extracellular volume, while feature tracking CMR allows accurate evaluation of myocardial deformation.  Table 1 summarises the strengths of CMR and how these compare with echocardiography.

This review provides an overview of the role of CMR in the assessment and diagnosis of cardiomyopathy related to two of the commonest forms of valvular heart disease, aortic stenosis (AS), and mitral regurgitation (MR).

## 2. Aortic Stenosis

According to the current guidelines, valvular intervention is recommended for symptomatic patients with severe AS and asymptomatic patients with evidence of LV decompensation as noted by EF <50% or elevated BNP/NT-pro-BNP level more than twice the upper limit of normal [4]. The assessment of the aortic stenosis severity is conventionally performed with echocardiographic two-dimensional (2D) and Doppler assessment, with the main parameters being the peak jet velocity, the transvalvular mean gradient, and the valve area (as obtained by the continuity equation), while the ventricular function is routinely conducted with the echocardiographic assessment of the ejection fraction [5, 6]. Left ventricular ejection fraction (LVEF) has been traditionally one of the important prognostic markers of the disease, as patients with early left ventricular decompensation as evidenced by EF <60% have an increased risk of mortality, even after valve replacement [7] leading the National Institute for Health and Care Excellence (NICE) in the UK to recommend consideration of intervention when the LVEF <60% when the decline is attributed to the AS in their initial guidance in March 2021 [8]. While echocardiography remains the gold standard technique for the assessment and grading of aortic stenosis, new techniques and methodologies have emerged for better and detailed assessment of the left ventricular myocardium with CMR having a central role.

Over recent years, however, assessment of myocardial deformation using strain imaging has been found to be of major significance in the evaluation of left ventricular function, especially for the large proportion of patients with aortic stenosis and preserved EF [9]. Several studies have suggested that global longitudinal strain (GLS) is of great prognostic importance and can detect subtle changes in LV function pre- and postaortic valve intervention, even with LVEF in the normal range [10]. Feature tracking (FT) CMR methodology is based on tracking the endocardial and epicardial borders of the ventricle on postprocessing of steady-state free precession (SSFP) cine images [11]. GLS obtained by FT CMR is a highly reproducible quantification technique that provides invaluable information about myocardial function without requiring any additional imaging acquisitions over and above the routinely acquired CMR images [12]. Evidence suggests that patients with asymptomatic severe AS and apparently normal LVEF do have in fact, impaired GLS, comparable to that of symptomatic patients with severe AS awaiting valve intervention [13]. This finding highlights the value of myocardial deformation analysis in this patient population. Abnormal GLS indicates the presence of potentially subclinical, impaired myocardial function, and that could help in the clinical decision-making process regarding the timing of intervention so as to avoid further potentially irreversible myocardial damage. Additionally, FT-derived GLS correlates well with the presence and extent of LGE, as strain values deteriorate when LGE is present [11, 14]. As the analysis of GLS is not dependent on gadolinium administration and does not require dedicated sequences (for example, when feature tracking is performed), it can therefore be assessed even in a noncontrast study during the postprocessing. This makes it a very useful measurement, specifically for the patients to whom MRI contrast agents may be contraindicated.

CMR is also the gold standard method for tissue characterisation as it allows the detection of changes of the myocardium on a cellular level [11]. In aortic stenosis, the left ventricular response to chronic pressure overload consists of an initial hypertrophic phase followed by cell apoptosis and eventually myocardial fibrosis [15]. Myocardial fibrosis is an early and fundamental part of this maladaptive response that determines changes in left ventricular function and heralds the presentation of symptoms and adverse events and therefore has prognostic implications [16].

Myocardial fibrosis in AS has a complex but characteristic pattern, formed mainly in two patterns which can be evaluated with the use of CMR, replacement (focal) and reactive (diffuse) fibrosis [17]. It has been demonstrated that both replacement and diffuse fibrosis are associated with the magnitude of LV hypertrophy, LV dysfunction, symptoms, and prognosis [18]. Interestingly enough, they are only weakly associated with the severity of the valve disease [18].

LGE is the paradigm method for the assessment of replacement (focal) fibrosis. Replacement fibrosis is the late phase of the disease process and represents cellular death that is followed by focal fibrosis (scar). This process is irreversible and is a strong predictor of adverse prognosis [19]. Replacement fibrosis can be detected and quantified with the use of CMR and LGE. This distinct pattern of midwall myocardial fibrosis signals the transition from the hypertrophic response to a decompensated phase with the occurrence of symptoms [15]. The poor prognosis associated with this irreversible stage persists even after aortic valve intervention and is strongly associated with all-cause and cardiovascular mortality [20]. Interestingly, this correlation appears to be “dose-dependent,” related to the scar burden. A large study conducted by the British Society for Cardiovascular Magnetic Resonance Valve Consortium, including six hundred seventy-four patients, demonstrated that for every 1% increase in myocardial scar burden, there was an 11% increase in all-cause mortality and an 8% increase in cardiovascular mortality [20].

While LGE is a well-established method for identifying replacement fibrosis, quantification can be challenging when diffuse fibrosis is present. The added value of T1 mapping-derived measurements enables the detection and quantification of reactive (diffuse) interstitial fibrosis, which is an early and importantly reversible stage of the disease process [11]. CMR with T1 mapping has the strength of differentiating changes on a cellular level from changes on an extracellular level. Given that reactive fibrosis represents the expansion of the extracellular matrix, T1 mapping-derived techniques can be used to assess diffuse fibrosis by quantifying the extracellular volume fraction (ECV%) and the indexed extracellular volume (iECV), which represents the total fibrosis burden [21, 22]. Combined with the LV mass, these parameters can provide a comprehensive assessment of LV remodelling regarding both the cellular and the extracellular compartments [19]. Since diffuse fibrosis is potentially reversible, the contribution of CMR is invaluable as thorough assessment and quantification of the diffuse fibrosis are essential to identify early decompensation and allow prompt intervention [22]. In a recent study by Everett et al. that included more than four hundred patients with severe aortic stenosis undergoing aortic valve replacement, diffuse fibrosis as assessed by T1 mapping was found to be an independent predictor of all-cause mortality [22]. Like the midwall fibrosis data, a dose dependency was also found, with every 1% increase in the ECV% followed by a 10% rise in the risk of death [22].

These findings have triggered questions in the scientific community about the appropriate timing of intervention and whether this should be driven by early markers of LV decompensation rather than the development of symptoms when irreversible damage may have possibly already occurred. The EVOLVED (Early Valve Replacement Guided by Biomarkers of Left Ventricular Decompensation in Asymptomatic Patients with Severe Aortic Stenosis) trial is a multicentre randomised controlled trial in which patients with asymptomatic severe (AS), LVEF ≥50%, and midwall LGE are randomised either to an early intervention or conventional “watch and wait” approach (NCT03094143) [23]. It aims in this way, to investigate whether an intervention based on objective evidence of early LV decompensation will be better for the patients clinically and prognostically.

**Figure 1:**
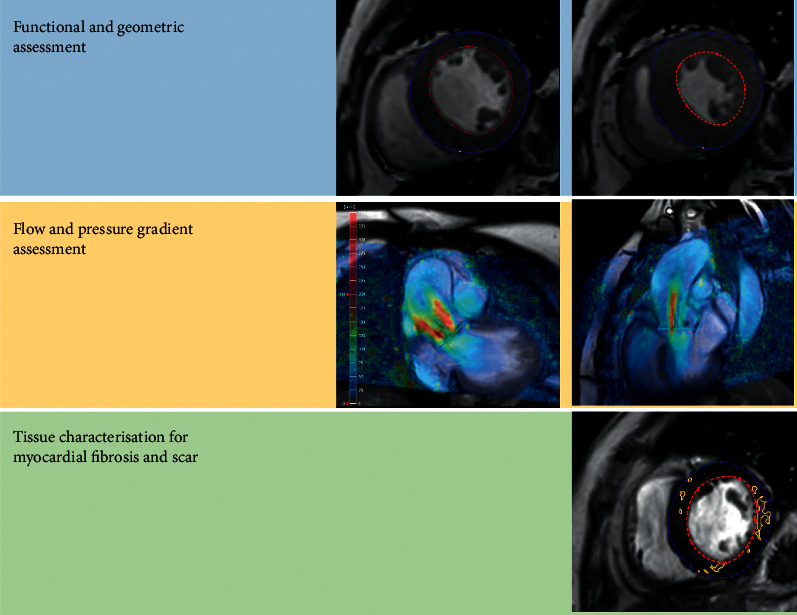
A case of severe aortic stenosis. (a) CMR assessment of left ventricular function and geometry. (b) 4D flow assessment of the severity of the aortic stenosis. (c) Tissue characterisation and assessment of myocardial fibrosis.

**Figure 2:**
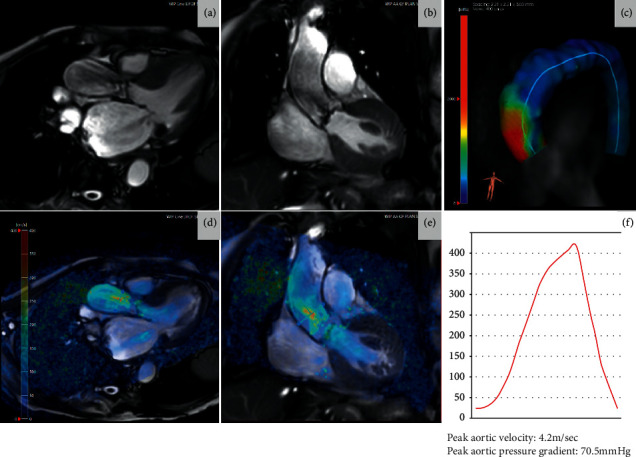
A case of aortic stenosis assessment using four-dimensional flow CMR. (a, b) Cine views demonstrating thickened and restrictive opening of aortic valve leaflets with flow acceleration in the aortic root. (c) Increased wall sheer stress on the anterior wall of the aortic sinus and ascending aorta due to eccentric jet through the stenosed valve. (d, e) Cine views with velocity overlay demonstrating flow acceleration greater than 4 m/sec and a two-dimensional plane through that to quantify peak velocity. (f) Peak velocity was consistent with severe aortic stenosis in this case.

Additionally, 4D flow is an emerging CMR tool that is becoming increasingly popular as an alternative tool for the assessment of aortic stenosis. This noninvasive tool can provide an accurate evaluation of the valvular lesion as the measurements acquired are not subject to the common errors and restrictions that accompany the two-dimensional echocardiography [24]. Furthermore, 4D flow CMR can provide invaluable information and comprehensive evaluation of aortic flow patterns and the haemodynamic consequences of severe aortic stenosis to the left ventricle and the aorta [25]. Figures 1 and 2 illustrate examples of CMR assessment of severe aortic stenosis with the CMR modalities discussed.

Undoubtedly, for patients with AS, CMR with both its conventional and emerging methods will continue to have a huge impact not only in diagnosis but also in prognosis, risk stratification, and potential decisions about the appropriate timing of intervention.

## 3. Mitral Regurgitation

Echocardiography is the primary investigation for the assessment of severity and mechanism of mitral regurgitation (MR). Transthoracic echocardiography may often be followed by a transoesophageal echocardiographic study for better assessment of the severity and mechanism of the mitral regurgitation. Standard 2D and Doppler echocardiographic methods of quantification of the severity include the vena contracta, proximal isovelocity surface area (PISA) method, and qualitative assessment of the continuous wave Doppler of the MR jet [26, 27]. Nevertheless, complex valvular anatomy or morphology or mechanism of regurgitation (multiple jets or very eccentric jets) may present a limitation of these techniques and may limit their diagnostic yield.

CMR has a central role in the evaluation of MR as it provides a comprehensive assessment of the valve anatomy, morphology, and accurate quantification of the MR [28]. MR can be either primary, where one or more structures of the valve apparatus are affected or secondary (functional), where regurgitation results from increased tethering forces stemming from either left ventricular or left atrial geometric alterations and subsequent annular dilatation [4]. CMR offers unlimited imaging planes that aid in the comprehensive assessment of the complex mitral valve apparatus and is an excellent tool that can be utilised if diagnostic uncertainty remains after echocardiography [29, 30]. This is reflected in clinical guidelines that suggest CMR assessment in patients where ventricular function and dimensions are insufficiently evaluated by echocardiography [4].

The haemodynamic impact of the mitral regurgitation on the myocardium is essential and traditionally this is evaluated with the 2D and volumetric assessment of the left ventricle and the left atrium [26, 27]. The LV end-diastolic and end-systolic volumes acquired from the standard volume quantification methods, together with forward flow measurements of the aorta or pulmonary artery, are used for the calculation of the regurgitant volume (MR regurgitant volume = LVSV–forward flow) and also determine the degree of LV dilatation resulting from the MR [28, 30]. In this way, the impact of chronic volume overload can be thoroughly assessed, and the haemodynamic effects of the mitral regurgitation on the left ventricle can be appreciated [31]. Current guidelines highlight the importance of LVEF and end-systolic cavity size in decision-making regarding the timing of intervention with LVEF ≤60% and LVESD ≥40 mm being markers of ventricular decompensation and worse outcomes, triggering, therefore, the referral for intervention regardless of symptoms [4]. There is, however, much debate regarding this matter, as, similar to aortic stenosis, there is evidence suggesting the occurrence of subtle myocardial impairment in the presence of normal LVEF and normal cavity size [31]. Such observations lead to a hypothesis in which LV remodelling, and indeed myocardial fibrosis, can occur before a decline in LVEF and even before the development of symptoms [11]. Thus, in a small study of 35 patients with asymptomatic primary moderate or severe MR, diffuse interstitial fibrosis as measured by ECV was noted to occur before the occurrence of conventional indications for valvular intervention [32]. This was correlated with impaired myocardial deformation and reduced exercise tolerance assessed by cardiopulmonary exercise testing [32]. This finding has been confirmed in another large study comprised of 120 patients with chronic primary MR, which demonstrated that fibrosis, as quantified by ECV, occurs before the onset of symptoms [33].

Whereas early studies suggest a diffuse interstitial fibrosis pattern, more recent studies indicate there may also be a coexistent focal replacement fibrosis model. Recently, Kitkugvan et al. investigated the pattern of fibrosis and whether diffuse interstitial fibrosis was the result or the cause of the MR in a study that included more than four hundred patients with chronic primary MR [34]. The presence of diffuse fibrosis and raised ECV correlated with the severity of MR and was independently associated with symptoms and clinical events that included mitral surgery and cardiovascular death [34]. While diffuse interstitial fibrosis with raised ECV was raised in a similar fashion in patients with and without MVP, it was noted that replacement fibrosis with increased LGE was more prevalent in the individuals with MVP [34]. This finding was in agreement with a further study that included four hundred patients with MVP of variable severity [35]. This found that replacement fibrosis is common in patients with MVP and is independently associated with adverse cardiovascular events [35].

Although the pattern and spectrum of fibrosis in MR remain complex, the consequences of it remain rather clear. More specifically, it has been found that in young and middle-aged patients with mitral valve prolapse (MVP), there is a clear association of replacement fibrosis, as assessed by LGE, with life-threatening arrhythmias and risk of sudden cardiac death [36]. The myocardial scarring in this patient population targets specific myocardial regions, including the papillary muscles and the inferobasal LV wall [36, 37]. The presence of this nonischaemic pattern of LGE that appears to be in high prevalence in patients with MVP is not directly associated with the severity of the MR but has been repeatedly found to be a substrate for arrhythmic events [36–38]. The negative prognostic value of the presence of fibrosis may persist even after the intervention, although further large studies are needed to confirm a causal relationship between LGE and adverse events [39].

**Figure 3:**
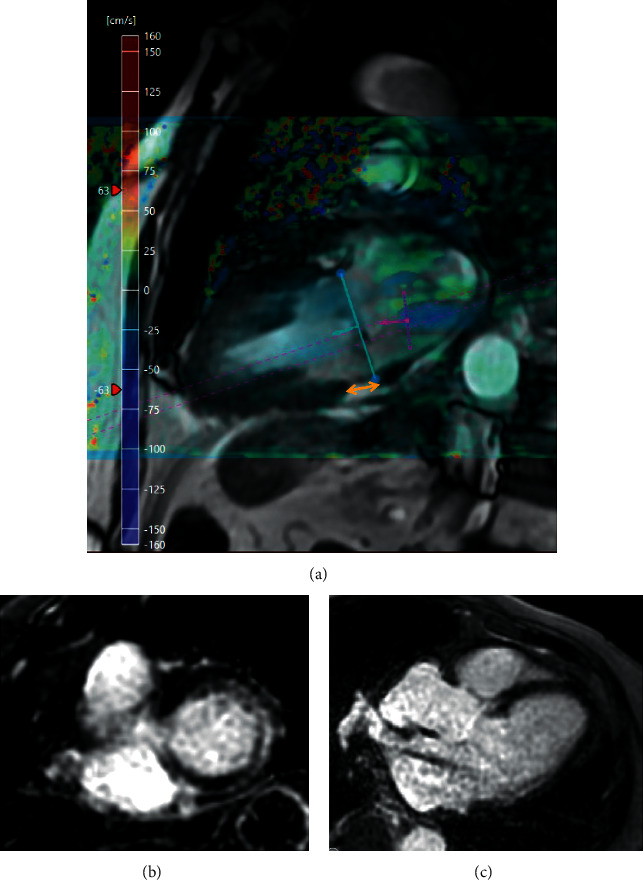
A case of mitral annular disjunction (a) (yellow arrow) with associated fibrosis in the basal lateral wall (b, c) (late gadolinium enhancement imaging) and moderate mitral regurgitation (a) (blue flow in the left atrium).

CMR has also contributed significantly to the identification of the entity of mitral annular disjunction as a common cause of arrhythmias [37]. Mitral annular disjunction is defined as an atrial displacement of the hinge point of the mitral valve away from the ventricular myocardium, leading to paradoxical haemodynamics and acting as an arrhythmic trigger [37, 40]. Whereas this pathological finding was thought to be linked with MVP, it has been demonstrated that the two are separate, although commonly coexistent entities [37, 40].  Figure 3 demonstrates the CMR assessment of a case of mitral annular disjunction with myocardial fibrosis and moderate mitral regurgitation.

**Figure 4:**
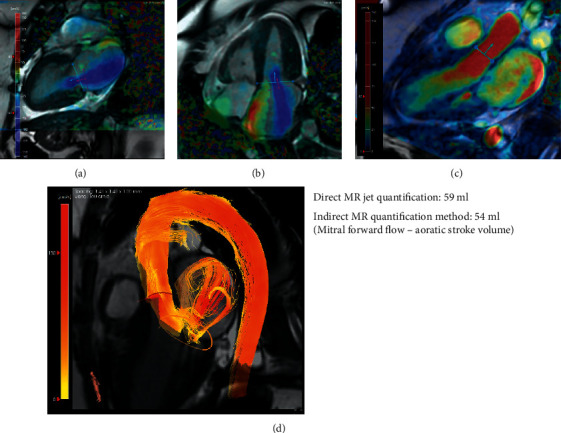
A case of mitral regurgitation assessment using four-dimensional flow CMR. (a) Two-chamber cine view with velocity overlay demonstrating mitral regurgitation (blue flow in the left atrium). (b) Four-chamber cine view shows the same mitral regurgitation jet which is swirling in the whole left atrium. (c) A three-chamber cine view used to quantify aortic stroke volume by valve tracking. (d) Three-dimensional streamlines of blood flow during systole demonstrating aortic forward flow and the mitral regurgitation swirling in the left atrium.

More recently, 4D flow CMR assessment and quantification of mitral valve regurgitation is increasingly used in clinical practice as it offers a much improved and detailed method of evaluation, especially for challenging cases, such as complicated anatomical lesions, multiple coexistent lesions or shunts [28]. The severity of the mitral regurgitation can be evaluated with 4D flow CMR either with direct quantification of the MR flow with retrospective mitral valve tracking or with indirect method, which is the mitral forward flow minus the aortic forward volume [28] (Figure 4). The exact mechanism and pathophysiology behind the findings may remain complicated and unclear, but CMR has undoubtedly the potential to support researchers in the quest to uncover the clinical and prognostic implications of the complex anatomical features and the presence of the different types of fibrosis.

## 4. Conclusions

The assessment and management of valvular heart disease have long centred on a triad of the presence or absence of symptoms, valvular functional parameters confirming severity, and the presence or absence of LV dilatation or dysfunction assessed by LVEF. Furthermore, the assessment of valvular function and LV dysfunction has long been dominated by echocardiography. The utility of CMR in VHD-related cardiomyopathy offers more accurate and incremental information than that derived from echo. The ability to image in any plane also offers structural analysis, particularly important in mitral valvular assessment. Through its delivery of accurate and highly reproducible chamber quantification CMR provides a gold standard for volumetric assessment, and in turn, myocardial mass measurement. Moreover, with LGE imaging and T1 mapping, CMR also offers deep tissue characterisation, which is emerging as a powerful technique in the identification of myocardial fibrosis with potential implications for risk stratification and timing of valvular intervention. Whether the timing of valvular intervention based on these early markers of LV decompensation results in improved clinical outcomes remains, however, to be determined and is the subject of active clinical trials.

## Figures and Tables

**Table 1:** Techniques available with CMR and comparison with echocardiography. Symbol “+” represents “good” and “++” represents “very good.” Symbol “−” is used when there is no means to assess the particular method.

Assessment methods	CMR	Echocardiography
Chamber quantification (wall thickness, mass, and volumes)	++	+
	The high spatial resolution allows accurate qualitative and quantitative assessment of cardiac chambers	Measurements are dependent on several parameters (acoustic windows, endocardial definition, on-axis/off-axis views, and sonographer)
Assessment of myocardial deformation (most commonly global longitudinal strain)	++	++
	Dedicated accurate sequences can be used for CMR strain. Furthermore, reproducible method of myocardial deformation assessment using feature tracking postprocessing of SSFP cine images	A reproducible method that provides valuable information, as long as certain requirements are fulfilled (clear endocardial definition and frame rate >50)
Comprehensive assessment of valvular anatomy and structure	++	+
	Unlimited imaging planes and high spatial resolution help in the detailed assessment of simple and complex valvular anatomy	Comprehensive anatomical assessment that is, however, limited in a certain number of imaging planes and by spatial resolution, which is lower compared to CMR
Qualitative and quantitative assessment of valvular lesions (regurgitation/stenosis)	++	++
	CMR is very useful in the assessment of the severity of valvular lesions that are difficult to be quantified with echocardiography (e.g., very eccentric jets)	High temporal resolution and assessment with colour and continuous wave Doppler offers a detailed evaluation of the severity of valvular lesions
Tissue characterisation (LGE and T1 mapping)	++	−
	CMR is the gold standard method for direct assessment of fibrosis with the use of LGE and T1 mapping techniques	Not available with echocardiography. Echocardiography backscatter can associate with myocardial fibrosis, but it is not an accurate method.

## Data Availability

No data were used to support this study.
